# Can prior hospitalisation experiences influence satisfaction with nursing care? Results in a school-aged children sample

**DOI:** 10.1080/07853890.2021.1895966

**Published:** 2021-09-28

**Authors:** Fernanda Loureiro, Zaida Charepe

**Affiliations:** a Escola Superior de Saúde Egas Moniz (ESSEM), Egas Moniz Cooperativa de Ensino Superior, Caparica, Portugal; b Universidade Católica Portuguesa, Instituto Ciências da Saúde, CIIS-Centro de Investigação Interdisciplinar em Saúde, UCP/ICS, Lisboa, Portugal

## Abstract

**Introduction:**

Patient satisfaction is considered an important and relevant patient outcome in the nursing field [1], and prior hospitalisation experiences have impact in the overall satisfaction with care [2]. Regarding children satisfaction, authors conceptualise satisfaction through the comparison of previous experiences [3]. This study aims to identify if prior hospitalisation experiences influences satisfaction with nursing care, in school-aged children (7–11 years).

**Materials and Methods:**

An observational, cross-sectional, exploratory-descriptive study with a convenience sample was performed. Data were collected from January 2015 to December 2016. The "Children Care Quality at Hospital" [4] instrument was used after translation and validation to Portuguese [5]. In this questionnaire children were asked to rate nursing care from 1(less satisfied) to 5 (more satisfied). Statistical analysis was performed using SPSS statistical tool (version 24.0). In order to verify the association between the variable’s prior to hospitalisation experience and patient satisfaction, Student's *t*-test was applied with a 95% confidence interval. Authorisation was obtained from ethics committees in each of the 6 hospitals were the study was applied, and also from the National Data Protection Commission.

**Results:**

In this sample (*n* = 252) children mean age was 8.9 years (SD = 1.4), and it mainly consisted of boys (52.8%, *n* = 133). Most children had prior hospitalisation experiences (63.5%, *n* = 160), 35.7% (*n* = 90) have never been hospitalised before, and 2 children answered, “I do not know”. Nursing care was rated with a score of 4.51 (SD = 0.645). There was no significant difference between having or not having prior hospitalisation experiences and the score attributed by children (*t* = 1.47; *p* =.821).

**Discussion and conclusions:**

In this sample, children are satisfied with nursing care provided during hospitalisation. In previous studies with adult population, prior experiences seem to have a negative effect on the overall satisfaction [2,6]. Specifically in school-aged children, previous experiences positively influences satisfaction with nursing care [4]. However, this was not verified in our study. We suggest that further studies should be developed some time after the hospitalisation experience, for example 6 months, to understand the most relevant experiences and their influence on the satisfaction with hospital nursing care.

## References

[1] Liu Y, Avant KC, Aungsuroch Y, et al. Patient outcomes in the field of nursing: a concept analysis. Int J Nurs Sci. 2014;1(1):69–74.

[2] Quintana JM, González N, Bilbao A, et al. Predictors of patient satisfaction with hospital health care. BMC Health Serv Res. 2006;6:102. 16914046 10.1186/1472-6963-6-102 PMC1579213

[3] Taylor RL, Olds T, Boshoff K, et al. Children’s conceptualization of the term “satisfaction”: relevance for measuring health outcomes. Child Care Health Dev. 2010;36:663–669. 20533914 10.1111/j.1365-2214.2010.01105.x

[4] Pelander T, Leino-Kilpi H, Katajisto J. Quality of pediatric nursing care in Finland. J Nurs Care Qual. 2007;22:185–194. 17353757 10.1097/01.NCQ.0000263110.38591.9a

[5] Loureiro FM, Araújo BR, Charepe ZB. Adaptation and validation of the instrument ‘Children Care Quality at Hospital ’ for Portuguese. Aquichan. 2019;19(4):1–13.

[6] Murante AM, Seghieri C, Brown A, et al. How do hospitalization experience and institutional characteristics influence inpatient satisfaction? A multilevel approach. Int J Health Plann Manage. 2014;29:e247–e260. 23818333 10.1002/hpm.2201 PMC4229067

